# Cryo-EM reveals a conserved baseplate architecture and distinct accessory protein assemblies in mycobacteriophages Claus, Corndog and Mysterious

**DOI:** 10.21203/rs.3.rs-9616548/v1

**Published:** 2026-06-05

**Authors:** Sunil Kumar Tewary, Chun-Hsiung Wang, Ming-Ching Lin, Melvin C. Shen, Li-An Tsai, Jitendra Maharana, Ronelito J. Perez, Chi-En Mou, Graham F. Hatfull, Todd L. Lowary, Meng-Chiao Ho

**Affiliations:** 1 Institute of Biological Chemistry, Academia Sinica, Taipei, Taiwan; 2 Institute of Biochemical Sciences, National Taiwan University, Taipei, Taiwan; 3 Department of Pharmacology and Toxicology, University of Toronto, Toronto, Canada; 4 Department of Biological Sciences, University of Pittsburgh, Pittsburgh, Pennsylvania, USA.

## Abstract

Mycobacteriophages, viruses that infect mycobacteria, hold promise as therapeutics against antibiotic-resistant mycobacterial infections. Although thousands of mycobacteriophages have been isolated, high-resolution baseplate structures have been reported for only two, revealing a knowledge gap in understanding phage–host interactions. Most mycobacteriophages are siphophages, possessing four structural components: capsid, connector, non-contractile tail and baseplate. The baseplate plays a critical role in infection by mediating host recognition, adsorption, and genome delivery. Here, we present atomic-resolution cryo-EM baseplate structures of three mycobacteriophages, Claus, Corndog, and Mysterious. These have inverted crown-, barrel-, and cage-like architectures, respectively, but despite their significantly different appearances, the baseplate cores possess a conserved architecture featuring a 6:6:3 stoichiometry of homohexameric tail tube proteins (TTPs), distal tail proteins (DTPs or Dits) and homotrimeric baseplate hub proteins (BHPs). The central core of the baseplate is formed by DTPs and BHPs, with the terminal TTPs making direct proximal contacts with the DTPs and contributing to the overall structure. While the core is morphologically conserved, some domains exhibit notable structural divergence across the three phages. Surrounding the conserved cores are diverse baseplate accessory proteins (BAPs), including upper baseplate proteins (BppUs), central fiber proteins (CFPs) and others, that assemble into distinct peripheral architectures and give rise to two baseplate-opening (or tail tube opening) mechanisms. These architectures reveal a conserved baseplate core complex diversified by BAPs, a feature that may be shared across mycobacteriophage siphophage families.

## Introduction

Mycobacteria are a diverse group of bacteria, ranging from highly pathogenic strains such as *Mycobacterium tuberculosis* to non-pathogenic environmental isolates. *M. tuberculosis* remains a major global health burden, causing tuberculosis (TB) and leading to more than a million deaths annually [1]. Additionally, non-tuberculosis mycobacteria (NTM), such as *Mycobacterium abscessus and Mycobacterium avium* complex, are opportunistic pathogens [2]. A defining feature of mycobacteria is their cell envelope, which is composed of a peptidoglycan backbone linked to arabinogalactan and further esterified with long-chain mycolic acids, elaborated with additional glycolipids and surrounded by a capsular layer. This dense barrier confers intrinsic resistance to many antibiotics and poses a formidable obstacle to antimicrobial treatment [3, 4].

Mycobacteriophages are viruses that specifically infect *Mycobacterium* spp. and have evolved mechanisms to recognize, attach and penetrate the complex mycobacterial cell envelope to deliver their genome into the host cytoplasm [5–8]. Recent clinical studies have demonstrated the therapeutic potential of mycobacteriophages in treating drug-resistant NTM infections, highlighting phage therapy as an alternative or complement to conventional antibiotics for treating mycobacterial diseases, including possibly tuberculosis [9–11].

Mycobacteriophage genomes are extraordinarily diverse and mosaic. Based on overall nucleotide sequence similarity, nearly 2800 sequenced mycobacteriophages have been sorted into 37 clusters (named A, B, C… AJ), along with six singletons that lack close relatives [12–14]. At the protein level, individual open reading frames (gene products) are grouped into ‘phamilies’ sharing greater than 27.5% amino acid sequence identity [15]. Over half of these phamilies contain only a single member, and most gene products belong to phamilies with three or fewer members [16] underscoring the extreme amino acid sequence variability of mycobacteriophage proteins.

This extensive genetic diversity is particularly intriguing given that the vast majority of mycobacteriophages are morphologically classified as siphophages, sharing a conserved overall virion architecture (an icosahedral capsid, a connector, a flexible non-contractile tail and a baseplate) [7, 8, 17]. Despite this apparent morphological conservation, proteins involved in virion assembly across different clusters often belong to different phamilies, sharing less than 25% sequence identity [16, 18]. These observations suggest that conserved architectural structures can be achieved through highly divergent molecular components, as has been demonstrated in capsid assembly [19]. Although some mycobacteriophages exhibit distinct yet overlapping host ranges, most infect the common laboratory host, *Mycobacterium smegmatis* [6, 20]. Notably, the host range of most mycobacteriophages has not been extensively characterized. At the distal end of the tail tube, the baseplate is a specialized multi-protein assembly that mediates host surface recognition, adsorption and initiation of genome delivery [7, 8]. AlphaFold modelling of individual mycobacteriophage baseplate proteins predicts markedly different structures [21]. However, the structural principles underlying this diversity, and the extent to which baseplate architecture is conserved or variable across clusters, or impacts host range, remain poorly understood.

Here, we present atomic-resolution cryo-EM structures of the baseplate complexes of three mycobacteriophages: Claus (2.63 Å resolution, Cluster L2), Corndog (2.86 Å resolution, Cluster O), and Mysterious (3.16 Å resolution, Cluster B5). Notably, the baseplate of Claus closely resembles that of Douge (Cluster L4) that we previously reported [7]. Claus and Douge exhibit relatively simple baseplate assemblies, each comprising six distinct proteins, whereas Corndog, previously reported Bxb1 (ClusterA1) and Mysterious display more complex and distinct architectures [8]. These structures reveal an intricate architectural organization that combines structural diversity with functional conservation across the baseplate assemblies. Our analyses reveal that, at least for these three mycobacteriophages as well as Douge and Bxb1, the baseplates follow a modular design principle, in which a structurally conserved architecture ensures basic core assembly and mediates the release of the tape measure protein upon host interaction [22, 23], while diversified accessory components may enable adaptation to various host surface features. By elucidating the structure, symmetry and intermolecular interactions within these complexes, this work lays a foundational framework for mycobacteriophage baseplate assembly and informs future efforts in phage engineering.

## Results

### Comprehensive architecture of the baseplate assembly in mycobacteriophages

**Figure 1.**
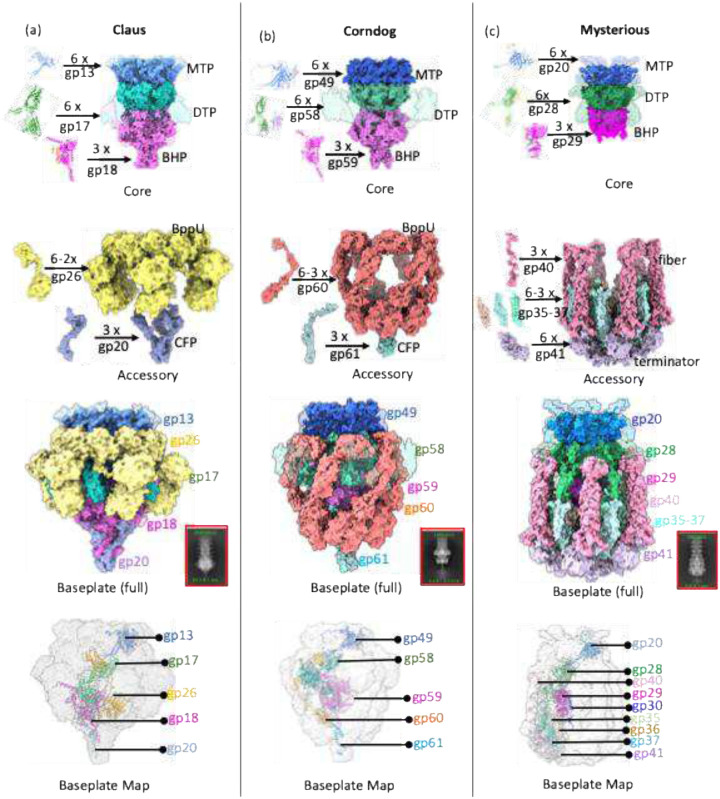
Structural comparison of mycobacteriophage baseplate assemblies. Structural assembly of (a) Claus, (b) Corndog and (c) Mysterious baseplates. The Claus Baseplate Core Complexes (top panel) consists of tail tube protein (TTP, blue), distal tail protein (DTP, green) and baseplate hub protein (BHP, magenta). Divergent regions of each monomeric core protomers are highlighted in lighter surface colors, whereas structurally conserved regions are shown as ribbon representations in same color scheme; divergent regions are shown in distinct colors. Individual TTP, DTP and BHP protomers are labelled with their gene name and oligomeric state. Second panel displayed the baseplate accessary proteins (BAPs): Oligomeric BppUs, and CFPs in Claus and Corndog; and the Mysterious shows all four fiber-like BAPs, gp35–37, gp40 and gp41 (baseplate terminator). Third panel shows the overall surface of Baseplate structure and corresponding 2D class averages, and bottom panel displays transparent cryo-EM map together with individual baseplate protomers denoted with corresponding gene product (gp) numbers.

Cryo-EM maps of baseplate complexes, also known as tail tip, from three purified mycobacteriophages, Claus (Cluster L2), Corndog (Cluster O), and Mysterious (Cluster B5), were reconstructed using a previously published method [7], achieving overall resolutions of 2.63 Å, 2.86 Å, and 3.16 Å respectively, with C3 symmetry imposed (Supplementary Figure 1 and Supplementary Table 1). Proteins involved in baseplate assembly were fully resolved in the cryo-EM map, except for the central fiber proteins (CFPs), of which only the N-terminal segments anchored to the baseplate were visible (summarized in Table 1 and Supplementary Figure 1–3). Notably, additional density attributable to the TMP is observed within the central lumen; however, this density was fragmented and diffuse, precluding reliable model building. Therefore, the TMP was not included in this study. Morphologically, the Claus baseplate, excluding the long tip, exhibits a radially symmetric, inverted crown shape reminiscent of bacteriophage Douge and *Escherichia coli* phage T5 [7, 23], while the Corndog and Bxb1 baseplates adopts a barrel-shaped assembly and Mysterious exhibits a more complicated cage-like architecture (Figure 1). These structural variations are attributed to differences in composition and structural diversity of the peripheral baseplate accessory proteins (BAPs). In Claus, a dodecameric gene product (gp), gp26 serves as the upper baseplate protein (BppU), while a trimeric gp20 forms the CFP (Figure 1a and Supplementary Figure 3). Corndog shares a similar architecture, with trimeric gp60 as the BppU and trimeric gp61 as the CFP (Figure 1b and Supplementary Figure 3). In contrast, Mysterious carries several BAPs to complete its baseplate, including trimeric gp40 and a heterotrimer composed of gp35, gp36 and gp37, all fiber-like shaped, as well as a hexameric gp41, and a trimeric gp30 as the CFP (Figure 1c and Supplementary Figure 3). Upon removal of the BAPs, a conserved baseplate core architecture is revealed in all three mycobacteriophages, indicating that the baseplate core is largely shielded by BAPs and therefore not solvent-exposed. This core, also known as the tail terminator [22], consists of a homohexameric distal tail protein (DTP), a homotrimeric baseplate hub protein (BHP) and a terminal layer of the homohexameric tail tube protein (TTP), which forms the tail tube, and interfaces with the DTP at the proximal side of the baseplate (Figure 1). All three core complexes adopt a 6:6:3 stoichiometry of TTP–DTP–BHP, characteristic of most structurally-reported siphophages [7, 8, 22]. Remarkably, the core architecture of these three baseplate assemblies remains conserved, despite the low sequence similarity among the constituent proteins (Figure 1 and Supplementary Table 2). Functionally, the baseplate core transforms the tail tube into an inverted cone, with the baseplate hub region functioning as a gating system to mediate TMP release and genome ejection upon host interaction [7, 8, 24, 25].

### Mycobacteriophage baseplate core TTP, DTP and BHP architecture

**Figure 2.**
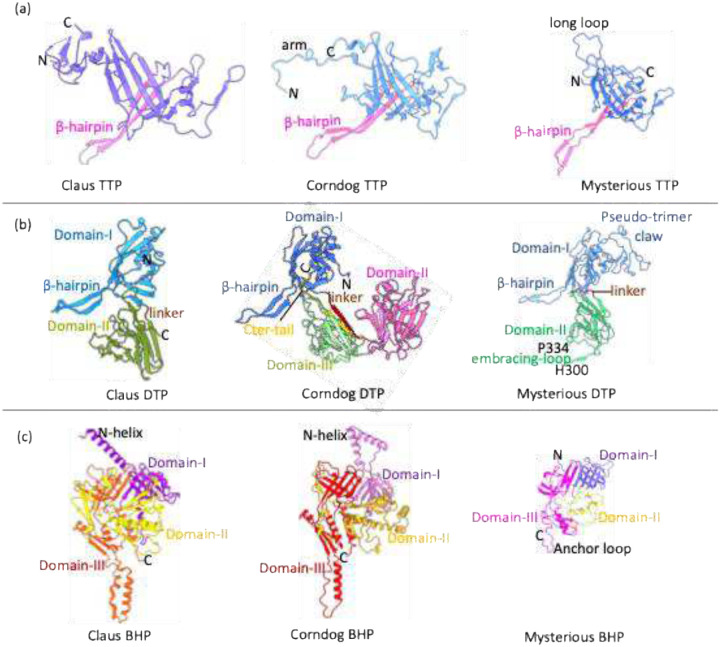
Structural comparison of Baseplate Core Complex components from mycobacteriophages Claus, Corndog, and Mysterious. (a) Representative protomers of the TTPs from Claus (gp13), Corndog (gp49) and Mysterious (gp20). Each TTP consists of β-sandwich-type fold. Additional structural features include small C-terminal domain II in Claus, an extended N-arm in Corndog and a knot-loop within Mysterious. (b) DTPs from Claus (gp17), Corndog (gp58) and Mysterious (gp28). Claus and Mysterious DTPs adopt two-domain architectures, whereas Corndog DTP contains three domains, including an extended domain III. (c) Baseplate hub proteins (BHPs) from Claus (gp18), Corndog (gp59) and Mysterious (gp29). All BHPs exhibit a tripartite architecture comprising an N-terminal β-rich domain (domain I), a central α/β-rich domain (domain II) and a C-terminal fiber-binding domain (domain III). In Claus and Corndog BHP, domain II features extended helices and β-sheets. In Mysterious BHP, an extended C-terminal anchor loop was observed. Representative protomers of the TTP, DTP, and BHP are color-coded to highlight the domain organization. N- and C- terminus are labeled.

The Claus, Corndog and Mysterious TTPs, similar to those in most reported phage structures, form homo-hexamer oligomers (Figure 1) [24–27]. Their TTP protomers exhibit a conserved structure fold with some minor differences (Figure 2a). The TTP protomer of Claus (gp13) has a β-sandwich-type fold flanked by an α-helix and a small C-terminal domain II (Figure 2a). Notably, the β-sandwich-type fold extends as a long β-hairpin with a loop at the tip, which acts as a mechanical hinge and helps TTP interactions with the DTP (discussed later). In Corndog, the TTP contains an additional long, unstructured N-terminal loop that extends outward as an arm, whereas in Mysterious a structurally distinct a long loop spanning residues 136–162 is observed in the central region of the protein (Figure 2a).

The DTPs in these three phages also exhibit conserved structural features characterized by a two-lobed architecture composed of two distinct domains. The N-terminal domain I features a canonical β-sandwich element flanked by an α-helix, while the C-terminal domain II adopts a distal β-sandwich (Figure 2b). A characteristic domain I feature that is conserved in Claus, Corndog and Mysterious is a surface-exposed β-hairpin motif, which is involved in interaction with the BHP. DTP domain I engages in inter-subunit interactions through hydrogen bonding and hydrophobic packing. Six neighboring domain I subunits assemble via interdigitated β-hairpins to form a ring-like hexameric complex. Meanwhile, domain II extends outward or downward, forming interfaces with the BHPs.

Among the three DTPs, the Claus DTP features the shortest inter-domain linker, composed of just two residues (E150 and P151), connecting domains I and II. Notably, the C-terminus of domain II in Claus also forms a short, anti-parallel, two-stranded β-sheet with domain I, further stabilizing the bi-domain orientation. In the Corndog DTP (gp58), the protein exhibits a three-lobed architecture due to the insertion of a Rossmann-like α/β fold protrusion as domain II (residues 198–416) between domain I (residues 1–188) and domain III (residues 417–583, equivalent domain II of Claus and Mysterious) (Figure 2b). A linker (residues 189–197) separates domain I from domain II and forms an extended β-sheet with domain III, possibly imposing conformational restraints between domains I and III. In the Mysterious DTP (gp28), domain I (residues 1–273) is separated from domain II (residues 290–462) by a flexible linker (residues 274–281), resulting in a different domain II orientation with an extended embracing loop (residues 293–345 modeled; however, the segment between residues 300–334 is not visible in the electron density map, suggesting structural disorder). This loop facilitates interaction with the gp35/36/37 heterotrimer (as part of the BAPs), which is absent in Claus and Corndog. Compared with the Claus and Corndog DTPs, domain I of the Mysterious DTP possesses an additional flexible loop that arranges three conserved arginine residues (R51, R67, R132) into a three-finger claw conformation, facilitating interaction with neighboring baseplate-associated proteins (discussed later).

In all the three phages, the homo-trimeric BHP situated below the hexameric DTPs (Figure 2c), transform the tubular structure into an inverted cone shape, thereby sealing the tail tube channel [7, 8, 25, 28, 29]. The BHP exhibits a three-domain architecture. Domain I (~150 residues) forms a β-sheet core, the extended β-core domain II (~200 residues) features two central helices flanked by two β-sheets on each side and domain III (~200 residues) adopts a compact β-sheet-rich lobe surrounded by an antiparallel two-stranded β-sheet and helices [5]. The β-sheets from domains I and III align antiparallel and side by side, forming an extended curved β-sheet. Three of these extended β-sheets assemble into a trimeric circular arrangement that directly interacts with the tubular DTPs on the top. Domain II is positioned below the extended β-sheet and, together with the extended β-sheet and helices from domain III, form an encapsulated vertex.

In both the Claus (gp18) and Corndog (gp59) BHPs, domain II features extended helices and β-sheets that give rise to a protruded vertex. Notably, the Mysterious BHP (gp29) is approximately 200 residues shorter than the Claus and Corndog BHPs, primarily due to the absence of the extended β-sheet and helices in domain III, as well as a more compact domain II. The C-terminal α-helices from the Claus and Corndog BHPs are tucked into the central lumen of the vertex, thereby stabilizing the trimeric structure with its inverted cone configuration (Figure 1a and Supplementary Figure 4 and 5). The trimeric Mysterious BHPs themselves cannot form a fully enclosed vertex and lack the protruded baseplate observed in both Claus and Corndog. Instead, in Mysterious, the N-terminal region of the trimeric CFPs insert to seal the vertex and also project the three helices into the central lumen of the vertex, thus resembling the intrinsic closed configuration observed in Claus and Corndog (discussed later).

### TTP–DTP and DTP–BHP baseplate core complex interactions

**Figure 3.**
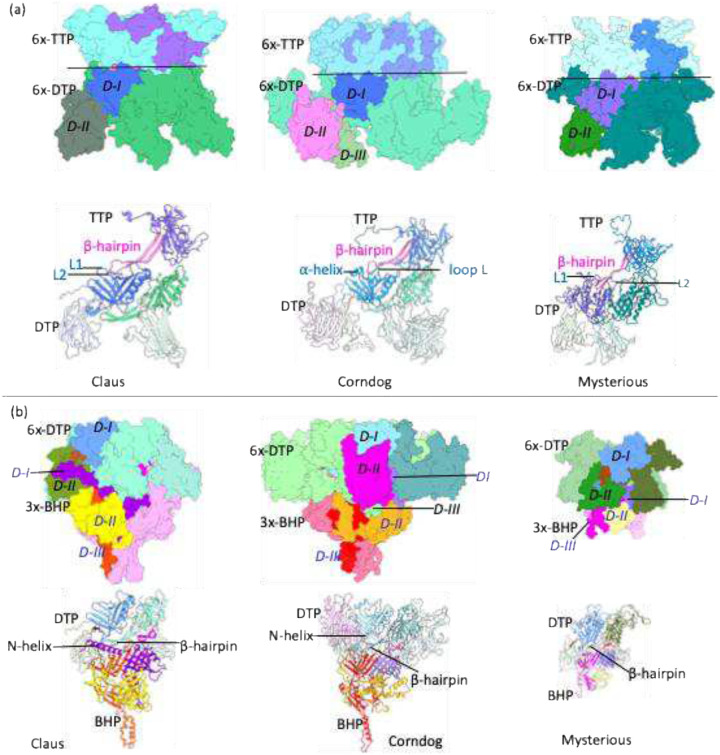
Comparative overview of conserved interactions of DTPs with TTPs and BHPs in Claus, Corndog and Mysterious Baseplates. (a) Structural comparison of TTP-DTP complexes from Claus, Corndog and Mysterious. Top panels show surface representations of the 1:1 arrangement of six TTP and six DTP subunits. Bottom panels show ribbon representations illustrating the 1:2 interface architecture with highlighted contact points. In all cases, a conserved TTP β-hairpin (pink) inserts between two DTP surfaces and forms the primary anchoring interaction. (b) Comparison of DTP–BHP complexes in Claus, Corndog, and Mysterious. Surface representations (top) show six DTP subunits bound to three BHP subunits, forming a 6:3 DTP–BHP assembly that links the distal tail to the baseplate. Ribbon representations (bottom) highlight the 2:1 interface architecture. DTP β-hairpins insert into grooves on the BHP protomer and provide the main anchoring contacts. Additional small interactions contributed from the third DTP β-hairpins are shown as red ribbon diagram. The DTP subunit interacting β-hairpin is shown in pink color for Claus, Corndog, and Mysterious and the and rest of the structure is hidden for clarity. Similarly DTP non interacting domains color is faded for clarity. One representative subunit of TTP, DTP, and BHP is color-coded consistently following Figure 2, to facilitate visualization of domain organization. Domains of DTPs are labelled with a black letter D while BHPs are blue on surface diagrams.

A conserved interaction pattern between the TTP and DTP is observed in Claus, Corndog, and Mysterious. Six TTP protomers assemble into a hexameric ring that interacts with a layer ring formed by hexameric DTP protomers, yielding a 6:6 TTP–DTP arrangement, in which each TTP engages two DTP protomers (Figure 3a). A protruding β-hairpin motif from each TTP extends downward and inserts into the surface of a DTP protomer positioned adjacent to the TTP below it. Notably, this insertion of the β-hairpin motif into the lower layer of the TTP ring is also conserved in inter-ring interaction within the tail tube. Although the overall binding mode is conserved, each mycobacteriophage shows distinct variations in the surrounding structural elements that engage with the β-hairpin motif, reflecting differences in the length and tilt angle of the β-hairpin. In Claus, the TTP β-hairpin engages the DTP together with two extended loops (L1 and L2) from domain I (Figure 3a). In Corndog, the loop L interaction is further supplemented by a short α-helix in the DTP that packs against the β-hairpin (Figure 3a). In contrast, in Mysterious, the L1/L2 loops adopt a slightly altered orientation to interact with the β-hairpin (Figure 3a). Together, these observations point to a unifying mechanism in which the TTP β-hairpin provides the primary anchor for DTP binding, while variations in loops, helices, and linker regions fine-tune the interface in a phage-specific manner. Additionally, beyond the β-hairpin regions (Figure 3a), the TTP also interacts extensively with domain I from the lower DTP, further reinforcing the TTP–DTP inter-ring interactions. All interactions are detailed in Supplementary Tables 3–5.

In Claus, Corndog, and Mysterious, as well as the two other structurally-characterized mycobacteriophages Bxb1 (Cluster A1) and Douge (Cluster L4), six DTP protomer subunits assemble into a homo-hexameric ring, engaging a trimer of the BHP [7, 8]. This results in a 6:3 DTP–BHP arrangement in which each BHP protomer contacts with three DTP protomers above it (Figures 1 and 3b). Similar to the TTPs, domain I of these DTPs possesses an extended β-hairpin that protrudes downward. Therefore, this inter-layer interaction between the DTP and BHP is anchored by multiple β-hairpins inserting into the surface grooves of the extended β-sheet formed by domains I and III of the BHPs. These β-hairpin insertions provide inter-locking interactions that likely secure the DTP–BHP interface. The linker between domains I and II of the DTP also engages the extended β-sheet of the BHPs, further reinforcing the inter-layer interactions. Addtionally, domain II of the Claus and Mysterious DTPs, and domain III of the Corndog DTPs, protrude downward and outward, laying across the upper surface of the β-sheet in the BHPs, forming multiple inter-protein contacts. In Claus and Corndog, this inter-layer interaction is further stabilized by the insertion of the N-terminal helix of the BHP into the DTP (Figure 3b). All interactions are detailed in Supplementary Tables 3–5.

### Assembly and interactions within the Claus BAPs

**Figure 4.**
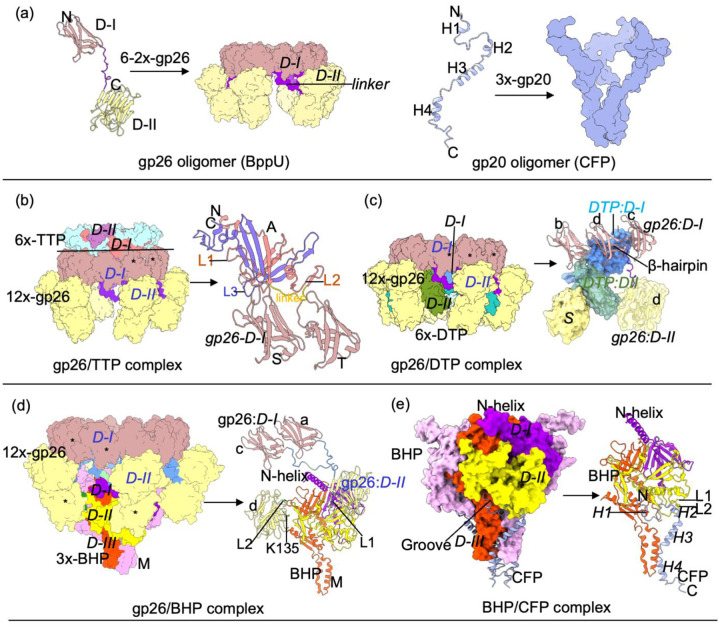
Structural organization and interaction network of Claus BAPs within the baseplate. (a) Domain architecture of gp26 (BppU) showing the N-terminal domain I (D-I, brown) connected by a short linker to the β-rich domain II (D-II, yellow). Gp26 assembles as a 12-subunit ring, positioning D-I toward the central axis. The Claus trimeric gp20 (CFP) is also shown, with helices (H1-H4) (b) Gp26–TTP complex: The TTP engages domain I of two gp26. For clarity, non-interacting domain II of gp26 is hidden. (c) Gp26–DTP complex: A hexameric DTP assembly (6 × DTP) engages multiple gp26 subunits. DTP domains are shown as D-I (blue surface) and D-II (green transparent surface), whereas gp26 domains are shown as D-I (brown) and D-II (yellow). The gp26 D-I β-hairpin and loop extensions insert into a groove on the DTP D-I domain, forming the primary anchoring interface. Additional lateral contacts are mediated by DTP D-II domain and gp26 D-II from neighboring subunits. (d) Interaction of gp26 with the BHP. Loops L1 and L2 from gp26 subunits a and d (green) interact with BHP domain II (deep yellow), while the linker of gp26 subunit c (blue) contacts the N-terminal helix of the BHP. An electrostatic interaction between gp26 K135 (subunit d, green stick) and BHP D305 further stabilizes the interface. (e) CFP–BHP complex: the trimeric CFP attaches beneath the BHP, with its N-terminal region inserted into the BHP Domain II (yellow) cavity, forming the distal attachment region of the baseplate. Domains of the TTP and DTP are labelled with a black letter D while gp26 is blue. The asterisk interacts the interacting domains of gp26. One representative subunit of the TTP, DTP, and BHP is color-coded consistently to facilitate visualization of domain organization.

The baseplate core complex associates with multiple BAPs, including the BppU, the CFP and others, to assemble structurally diverse baseplate architectures. Because the BAPs are more solvent-exposed and certain domains exhibit structural folds resembling those of carbohydrate-binding modules (CBMs), the BAPs are likely to engage in interactions with host cell wall glycans, consistent with our previous study showing that the BAPs of mycobacteriophage Douge interact with *M. smegmatis* surface glycans. Morphologically, the Claus baseplate has the simplest architecture among the three mycobacteriophages in this study, containing two BAPs: gp26 and gp20. The gp26 (313 amino acids) of Claus is the BppU, which forms a ring structure that attaches to the DTP [30, 31] and exhibits a two-domain structure (Figure 4a). The N-terminal domain I of the Claus BppU (residues 1–101) forms a compact Ig-like β-sandwich (Figure 4a) and is connected with a β-rich domain II (residues 119–313) whose structural fold closely resembles that of a CBM from *Bacillus subtilis* (PDB ID: 4B1M) [32]. This similarity suggests that the gp26 domain II may function as a glycan binding domain, potentially playing a role in host recognition. The gp26 domain I forms a dodecameric ring around the TTP and DTP, while domain II adopts two distinct orientations and forms a hexamer of dimers.

Two copies of the gp26 domain I engage one TTP protomer via loops (L1–L3) and a short β-sheet (Figure 4b). These interactions primarily consist of hydrogen bonds, with a few salt bridges, alongside some electrostatic and hydrophobic contributions (Supplementary Table 3). The DTP engages gp26 through two spatially and functionally distinct interfaces mediated by its domain I and domain II. The DTP domain I docks against domain I of gp26 primarily via the extended β-hairpin and loops protruding from gp26 that insert into a complementary groove on the DTP (Figure 4c). This interaction is dominated by electrostatic, hydrogen bonding and hydrophobic contacts at the interface, which anchor gp26 to the DTP scaffold (Supplementary Table 3). In contrast, the DTP domain II forms extensive lateral interactions with domain II of neighboring gp26 proteins, which project outward (Figure 4c). These interactions involve a broader surface area and are mediated by a combination of polar and hydrophobic contacts, contributing to the overall stability and multivalent nature of the gp26–DTP complex. The arrangement allows each DTP protomer to simultaneously coordinate multiple gp26 molecules, consistent with the observed six protomer DTP assembly engaging three gp26 subunits. Together, these interactions create a bipartite binding mode in which the interface between domain I of the TTP and domain I of gp26 provide precise geometric anchoring, while the interaction between domain II of the same two proteins enhances structural integrity and assembly cooperativity. This modular interaction strategy likely facilitates robust coupling between the DTP and gp26 (Supplementary Table 3).

In the gp26–BHP complex, gp26 forms a bridge between the tripodal BHP trimer and the outer TTP/DTP layers (Figure 4d). The gp26 linker inserts into clefts of the BHP protomer, while the gp26 peripheral domain II loops L1 and L2 provide stabilizing side-to-side contacts (Figure 4d). Notably, electrostatic interactions between K135 (gp26) and D305 (BHP) further stabilize the complex (Supplementary Table 3). Due to the extended structure of the CFP (gp20), cryo-EM could only resolve 69 residues at the N-terminus (Figure 4a, and Supplementary Figure 2a). The secondary structure identifies four short α-helices per chain (H1, residues 7–10; H2, residues 22–27; H3, residues 30–44; H4, residues 49–57). A coiled-coil region after the fourth α-helix packs as a trimeric helical bundle (Figure 4a). The CFP trimer docks beneath the BHP via the very beginning of the H1–H4 helices. At this interface, loops L1 and L2, and β-hairpin elements from the BHP domain I, interdigitate with the CFP helices to form a tight, complementary interaction surface.

Morphologically, the CFP adopts a three finger-like claw that clamps around the inverted cone-shaped BHP. All detailed interactions are listed in Supplementary Table 3. In the Claus baseplate, the interaction between the BHP (gp18) and the CFP (gp20) is mediated by a composite interface formed by multiple hydrophobic patches reinforced by specific polar anchors. Three discrete hydrophobic patches on the BHP contribute to CFP engagement (Supplementary Figure 6). Patch I is a dense aromatic–aliphatic cluster (F150, F158, F162, M163, L164, and A165) that forms a hydrophobic core at the interface, while patch II (M174, L178, L190, P194, and F228) further extends this contact surface along the periphery. A smaller patch III near the C-terminal region (A464–A465–L466) contributes additional hydrophobic stabilization. Notably, π–π stacking interactions involving aromatic residues, together with strategically positioned hydrogen-bonding contacts, may act as anchoring points that lock the CFP into a shallow groove on the BHP surface. Electrostatic surface complementarity and shape matching further promote tight packing of the interface. Collectively, these interactions generate a robust and highly ordered BHP–CFP assembly, highlighting how hydrophobic clustering combined with aromatic stacking and hydrogen-bond anchors underpin the stability of the Claus BHP–CFP interface (Supplementary Figure 5a and 6 and Supplementary Table 3).

### Assembly and interactions within the Corndog BAPs

**Figure 5.**
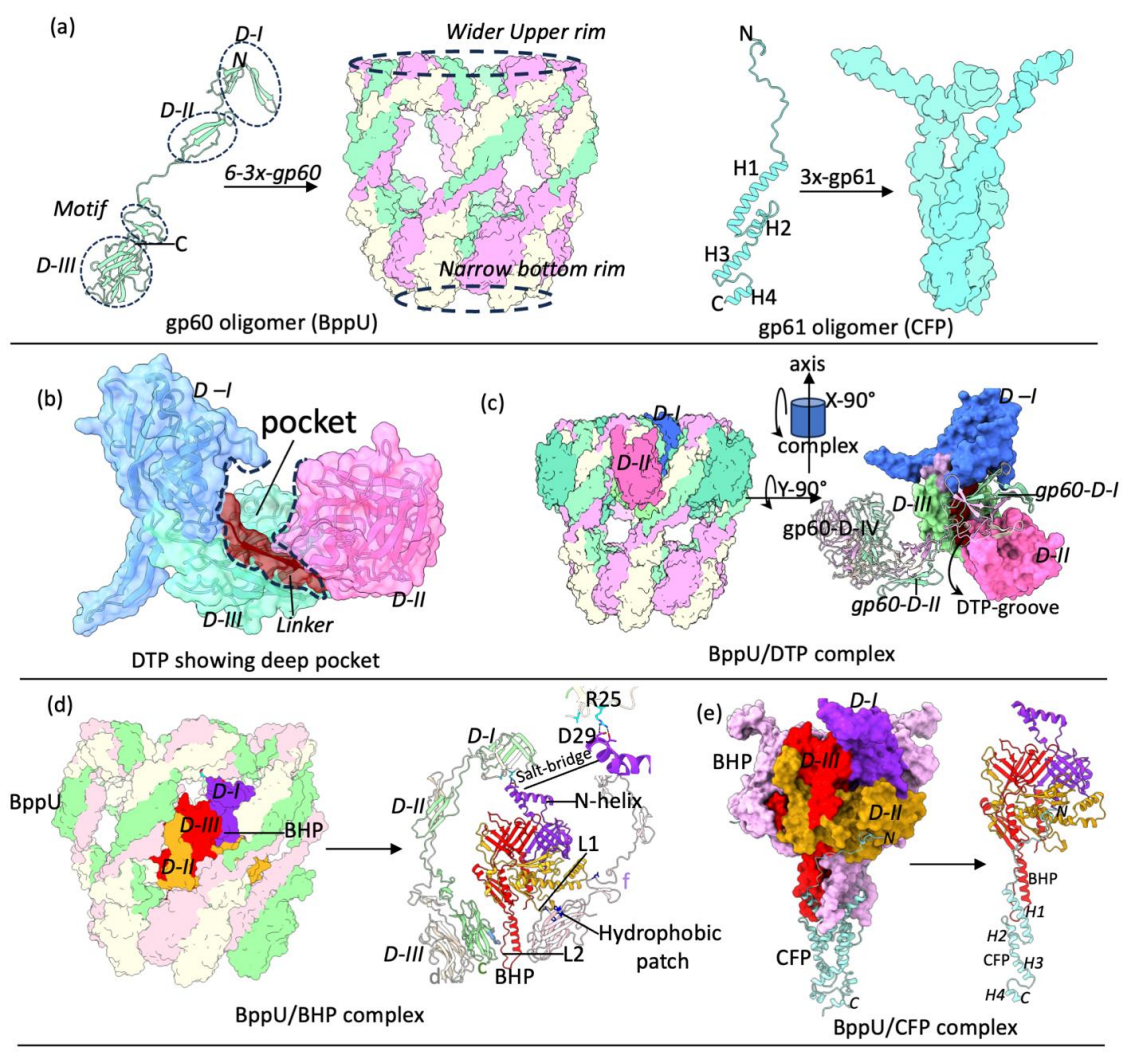
Structural organization of Corndog BAPs and its interaction within baseplate. (a) Domain architecture and assembly overview of Corndog gp60 (BppU). The gp60 monomer show three domains (D-I, D-II, and D-III) and a motif. Individual gp60 subunits assemble into elongated, crescent-shaped trimers with pronounced curvature. Gp60 subunits assemble into curved trimers that associate into bowl-shaped hexamers and further oligomerize into an 18-subunit ring with a widened upper rim and a constricted lower rim (left panel) and the trimeric gp61 (CFP) adopts a four-helix bundle. (b) Structure of DTP. Domain I (D-I, blue) and the protruding domain II (D-II, pink) form the walls of central cavity, whereas the C-terminal domain III (D-III, green) and connecting linker form base (c) Gp60–DTP complex. D-I trimers snugly fit into the DTP cavity stabilizing the complex. (d) Gp60–BHP complex. Domain I and III of gp60 interact with the BHP. BHP-L2 engages domain IV of two gp60 subunits, and a hydrophobic patch in domain-III of gp60 subunit f interacts with BHP loop L1. A zoomed-in view highlighting the salt bridge between BHP D29 and gp60 R25 is shown in the top-right corner. (e) CFP–BHP complex. The trimeric CFP (gp61) binds beneath the gp60–BHP layer. The loop–helix of BHP interacts with the helical stalk of CFP (helices H1–H4), which projects distally as a connector element. One representative subunit of the TTP, DTP, and BHP are color-coded following Figure 2 to facilitate visualization of domain organization.

Similar to Claus, Corndog also possesses two BAPs: gp60 is the BppU and gp61is the CFP (Figures 1b and 5). The gp60, a 281 amino acid protein, forms a homotrimer unit and each trimer binds to one DTP protomer, resulting in six copies of homotrimeric gp60 attached to the baseplate core complex (Figure 5a). Unlike Claus, no contacts of the Corndog gp60 are observed with its TTP. Each individual gp60 protein possesses 16 short β-strand segments and only two very short helices at amino acids **~**112–114 and ~269–271 (Figure 5a). In trimeric form, these β-strands cluster together, forming four β-rich domains separated by short linkers: domain I, residues ~1–46; domain II, residues ~58–100; small motif, residue ~122–145; and a larger C-terminal domain III, residues ~157–281 (Figure 5a). The short helix at residues ~112–114 sits at the domain II and motif III junction, and the helix at residues ~269–271 lies within the C-terminal portion of domain IV. The protein assembles hierarchically, with individual subunits forming elongated, crescent-shaped trimers that exhibit pronounced curvature. These trimers further associate into a hexamer, which oligomerizes to generate an 18-mer ring architecture characterized by a widened upper rim and a constricted lower rim (Figure 5a). The subunits are held together mainly by hydrophobic interactions and hydrogen bonds, with a few salt bridges contributing to stability (Supplementary Table 4). Each protein contributes its folded domains to the outer edge of the upper rim, while the short linker regions provide limited flexibility to allow smooth packing. Neighboring subunits interact through flat surfaces and flexible loops, forming a continuous ring. The Corndog DTP forms a deep pocket lined by domain I, central domain II, and the C-terminal domain III β-sheet/helix. (Figure 5b). The gp60 domain I trimer snugly fits into this deep pocket, thus anchoring the upper rim tightly. This snug interaction is stabilized by multivalent anchoring connections (Figure 5c and Supplementary Table 4). Overall, the gp60 may behave as rigid units that pack side-by-side, while flexible linkers accommodate curvature and enable formation of a closed disc that caps the baseplate, similar to what has been observed in the upper baseplate rings of *Lactococcus* phage TP901–1 [33]. The C-terminal domain of the gp60 also shares structural similarity with a lectin known to bind mannosides (PDB ID: 6TID) [34]. The barrel-like architecture of the Corndog baseplate, arising from the spatial arrangement of six gp60 homotrimers, resembles that reported for Bxb1. However, in Bxb1, this morphology is shaped by three additional CTE-like domains in the DTP, which differs markedly in its structural organization and assembly compared to that of Corndog.

Shown in Figure 5d are the Corndog gp60–BHP interactions, which are mediated by a combination of hydrophobic contacts, hydrogen bonding, and a salt bridge. One BHP engages with three subunits of the gp60, primarily engaging with domains I and IV of the gp60. A prominent hydrophobic patch involving gp60 helices and loops (L1 and L2) packs against complementary surfaces on the gp60, providing the major stabilizing force (Supplementary Table 4). Additional specificity is contributed by hydrogen bonds formed between flexible loop regions and adjacent secondary structure elements. A salt bridge, including interactions involving the BHP N-terminal helix containing charged residue D29 interacts with gp60 R25, further reinforces the complex (Figure 5d). Overall, the gp60–BHP interface is dominated by hydrophobic and hydrogen-bonding interactions, with salt bridges playing a minor but supportive role in stabilizing the assembly.

Similar to Claus, the Corndog CFP (gp61) appears flexible and only the N-terminal 103 residues can be observed in the cryo-EM map (Figure 5a and Supplementary Figure 2b). These 103 residues are in α-helical conformation and are organized into four short helices: H1, residues 33–56, H2, residues 67–76, H3, residues 78–90, and H4, residues 97–103. The CFP forms a trimer by coiled-coil interaction via the helical register (H1) and tandem short helices (H2 and H4), and attaches beneath the BppU–BHP layer via its helical stalk (Figure 5e). The interaction between the Corndog BHP and CFP is stabilized by a network of hydrophobic, aromatic, and electrostatic interactions that lock the BHP–CFP interface together (Supplementary Figure 7 and Supplementary Table 4). Three distinct hydrophobic patches on the BHP contribute to binding, with hydrophobic Patch I (W156, W157, L158 and F165,) forming the primary contact surface, Patch II (W283 and F287) reinforcing the interface through aromatic stacking, and Patch III (I169, I171, I471 and Y469) providing additional hydrophobic packing. The aromatic residues engage in π–π and hydrophobic interactions. In addition, specific salt bridges and hydrogen bonds, particularly involving the N-terminal region of the CFP and charged residues on the BHP, further stabilize the complex. Together, these interactions form a cooperative network that secures the CFP within a hydrophobic groove on the BHP, providing a tightly locked BHP–CFP assembly (Supplementary Figure 5b and 7). All detailed interactions are listed in Supplementary Table 4.

### Assembly and interactions of the Mysterious BAPs

**Figure 6.**
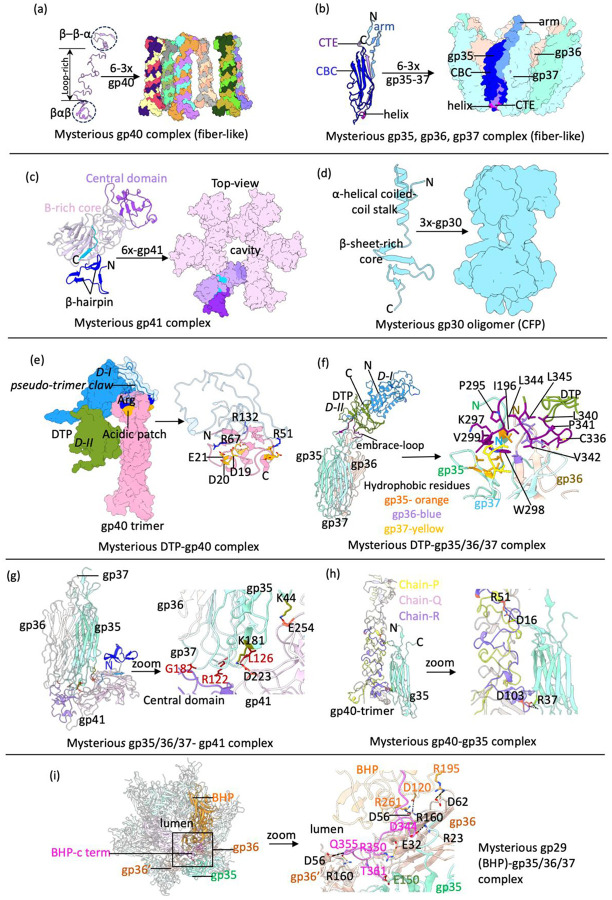
Structural organization and interaction network of Mysterious BAPs. Structural models of Mysterious baseplate components reveal the structural features different baseplate accessory proteins and their mode of interactions with the core proteins (DTP and BHP). (a) gp40-fiber like complex: gp40 protomer adopts a fiber-like fold with an N-terminal β–β–α motif, a loop-rich region, and a C-terminal β–α–β arrangement. Three protomers form a trimer and six trimers assemble into an 18-subunit tubular complex. (b) gp35–gp36–gp37 fiber-like complex: gp35 contains an N-terminal arm (light blue), central β-core (blue) and C-terminal extension (purple); gp36 cross-braces gp35–gp37. (c) gp41 hexameric complex: protomer contributes an N-terminal β-hairpin (blue) and β-rich core to form a toroidal ring (light purple) with a central segment (purple) forming the cavity, and a terminating C-terminal β-strand (cyan). (d) gp30 (CFP) trimer: an α-helical coiled–coil stalk capped by a β-core, stabilized by hydrophobic packing (left panel); the trimer is shown as a surface (right panel) (e) Gp40–DTP interaction: acidic gp40 residues (D19, E21) interact with basic DTP pseudo-trimer loop residues (R51, R67, R132). (f) gp35/gp36/gp37–DTP complex: extended loops (purple) from the DTP interlock with gp35/36/37 trimer grooves. The DTP K297-W298-V299 β-sheet pair with the gp35 N-terminal β-sheet and provide more stability. Hydrophobic residues of DTP loops are labelled, while the gp35/36/37 residues are shown in orange, blue and yellow, respectively. (g) gp35/gp36/gp37–gp41 complex: gp41 primarily contacts gp35 via loop-mediated electrostatic interactions (gp35 K44/K181 with gp41 E254/D223), with minor contacts from gp37; gp36 mainly stabilizes gp35. (h) gp40–gp35 complex: stabilized by two terminal salt bridges (gp40 R51-gp35 D16 and gp40 D103-gp35 R37) with additional hydrogen bonds and hydrophobic contacts. (i) Gp35/36/37–BHP interactions: multivalent contacts bridge adjacent heterotrimers through hydrogen bonds, electrostatic interactions, and salt bridges, positioning the BHP C-terminus as an interfacial clamp stabilizing the gp35/36/37 assembly. Yellow dashed lines denote the electrostatic contacts, while salt bridge and H-bonds are shown in black dashed lines.

Mysterious exhibits a more complex baseplate, composed six types of BAPs, including the homo-trimeric gp40, a heterotrimeric complex composed of gp35, gp36 and gp37, the homo-hexameric gp41, and homo-trimeric gp30 as the CFP (Figures 1c and 6a–d). The gp40 monomer has three regions: the N-terminal segment (residues 1–29), a long central segment (residues 14–119) and C-terminal region (residues 120–136) (Figure 6a). Three gp40 monomers intertwine into a trimeric fiber-like architecture and one homotrimer interacts with one DTP protomer, resulting in six homotrimers in the baseplate assembly (Figures 1c and 6a). The N-terminal segment adopts a structure with two short β-sheets followed by an α-helix. The tandem β-sheets are composed primarily of glycine, serine and small aliphatic residues, whereas acidic residues, including D19, D20 and E21 within the α-helix, form complementary electrostatic interactions with arginine residues R51, R67 and R132 of the corresponding DTP (Figure 6e). The central segment adopts a loop-rich architecture (Figure 6a) and the C-terminal region folds into a compact βαβ topology (Figure 6a).

Protomer gp35 is organized into three modules: an N-terminal projecting β-hairpin arm (residues 12–37), a central β-sheet sandwich core (residues 52–169), and a C-terminal extension (residues 170–205) containing a short helix (residues 179–182). Protomer gp36 adopts a similar β-strand-dominated architecture. Protomer gp37 displays a prominent elongated β-hairpin and an extended C-terminal segment that bridges gp35 and gp36. Together, gp35, gp36, and gp37 form a heterotrimer composed of a β-sandwich-like domain. Furthermore, six heterotrimers assemble into a hexamer via a β-sandwich-like domain and a N-terminal projecting β-hairpin arm, resulting in a bowl-shaped architecture with a central cavity (Figure 6b and Supplementary Figure 8).

Another BAP, gp41, with 378 residues, is dominated by β-sheets. The N-terminal region of gp41 adopts a triangular, β-rich fold in which three short β-strands define the sides of the triangle. Each vertex is accentuated by an extended loop or β-hairpin element, forming pronounced protrusions that project outward from the core and contribute to the overall three-lobed geometry (Figure 6c). Six gp41 protomers oligomerize into a pinwheel-like structure with a central cavity (Figure 6c). The central segment of gp41 contacts adjacent protomers and forms a ring inside, whereas six outward-facing β-sandwich domains protrude outward with its N-terminal triangular fold interacting with the gp40 trimeric fiber (Supplementary Figure 9a). The gp40 directly engages gp41 through a negatively-charged cleft centered at its C-terminal segment (Supplementary Figure 9a). The N-terminus of gp41 inserts into a cleft in the gp40 trimer; residues R7 and R18 of gp40 form salt bridges with gp41 D132. Additionally, gp40 R28 forms electrostatic contacts with complementary patch on gp41 (S111/Y115/D132) (Supplementary Figure 9a). Furthermore, Y134 from the gp40 trimeric fiber stabilizes the complex through aromatic and polar interactions (Supplementary Table 5). Together, hexameric gp41 and six copies of the gp40 trimeric fibers form a hexagon tube-like structure (Supplementary Figure 9b). One of the most structurally interesting interactions is between the Mysterious DTP and gp40 (Figure 6e). The DTP pseudo-trimeric claw (residues 1–136, part of domain I) forms a flexible, loop-rich structure that positions three conserved arginine residues (R51, R67, R132) in a claw-like arrangement (Figure 6e). Opposite to this, the gp40 trimer displays an acidic patch (D19, D20, E21), which establishes salt bridges with the DTP R51 and R67, while R132 forms electrostatic interactions. These electrostatic contacts stabilize the interface, with each arm of the claw engaging the acidic patch (Figure 6e). This charge-complementary interface resembles a ball-and-socket arrangement, where the acidic patch (gp40) docks into the arginine-rich claw (DTP). In addition, many hydrophobic interactions further stabilize the complex. As a result, the DTP, gp40 and gp41 form a cage encapsulating the rest of baseplate component. Notably, the spatial organization and ring assembly of gp41 resemble those of the receptor binding protein (RBP) in phage 80α, which recognizes wall teichoic acid in Gram-positive *Staphylococcus aureus* [35].

While DTP domain I interacts with gp40 as described above, DTP domain II (residues 293–345) engages the N-terminal projecting β-hairpin arm of the gp35/36/37 heterotrimer through an extended embracing loop (Figure 6f). This loop inserts into the N-terminal trimeric groove formed by gp35, gp36, and gp37, generating a clamping interaction that positions and stabilizes the six vertices of the hexagonal gp35/36/37 trimer assembly. For example, DTP residues P295, I296, W298, and V299 form a hydrophobic patch that packs against the hydrophobic groove, while the C-terminal motif (residues 336–345) further stabilizes this interface. Hydrogen bonds are also observed in the interface (Figure 6f). Additionally, DTP residues K297, W298 and V299 form a short β-strand that pairs in parallel with an N-terminal β-strand of gp35, providing directional stabilization. Together, these interactions lock the DTP loop into a composite groove formed by gp35, gp36, and gp37 (Supplementary Figure 10).

The Mysterious gp35/gp36/gp37 complex with gp41 resembles a bowl with a central cavity sitting on a hexagonal ring and channel passing through it. The gp41 interacts extensively with gp35 through forming a salt bridge between gp35 loops containing K44/K181 and E254/D223 of the β-sheet-rich core of gp41 (Figure 6g). In addition to these, there are several hydrogen bonds and a few hydrophobic interactions stabilizing the complex. The gp37 loops containing R122/L126/G182 provide minor contacts with gp41 (Figure 6g). The gp36 associates mainly with gp35, contributing to the elongation of the complex, but lacks direct interactions with gp41.

Analysis reveals that gp40 engages gp35 via two prominent salt bridges at opposite ends of the interface: an N-terminal salt bridge between gp40 Arg51 and gp35 Asp16, and a C-terminal salt bridge between gp40 Asp103 and gp35 Arg37 (Figure 6h). Moreover, the gp35–gp40 interface is reinforced by several hydrogen bonds and localized hydrophobic contacts.

The C-terminal region of the Mysterious BHP protrudes from the baseplate core and extends outward to form a distinctive anchor loop that latches onto an interfacial cavity created between two adjacent gp35/36/37 heterotrimers (Figure 6i). This positioning places the BHP C-terminus outside the central cavity of the baseplate assembly. Structurally, the anchor loop adopts a U-shaped conformation at its distal end, descending along the outer surface of the complex and engaging predominantly with gp36 and gp36′ subunits from neighboring heterotrimers, while forming comparatively fewer contacts with gp35 (Figure 6i and Supplementary Figure 11a). Through this configuration, the BHP C-terminal anchor loop functions as a clamping shaft that secures adjacent trimeric units and reinforces inter-trimer connectivity. The asymmetric nature of these interactions, which are dominated by gp36 contacts, indicates that the BHP C-terminus plays a stabilizing and coordinating role in maintaining overall baseplate integrity, primarily mediated by electrostatic interactions (Supplementary Figure 11b). As shown in Figure 6i, the BHP C-terminal region inserts into the interface between two neighboring gp35/36/37 heterotrimers, establishing an extensive network of stabilizing interactions across subunit boundaries. Rather than engaging in a one-to-one binding mode, the BHP C-terminus bridges two adjacent heterotrimers, interacting simultaneously with two gp36 protomers and one gp35 protomer, thereby reinforcing the heterotrimer–heterotrimer interface. Specifically, BHP residues D344, R350, Q355, and T361 participate in hydrogen bonding and electrostatic interactions with gp36 residues R23, E32, D56, and R160, as well as with gp35 residue E150, together forming a composite interfacial binding pocket. In addition, BHP residues R195, D120, and R261 form stabilizing salt bridges with gp36 residues D56, D62, and R160. Collectively, this multivalent interaction mode positions the BHP C-terminus as an interfacial clamp that stabilizes the gp35/36/37 assembly, supporting a structural anchoring role rather than a conventional one-to-one binding interaction. All detailed interactions involved in the BAPs are listed in Supplementary Table 5.

Like Claus and Corndog, our cryo-EM map can only reveal a portion of the Mysterious CFP due to the long flexible extended structure. In the case of Mysterious, only the N-terminal 64 residues of the CFP (gp30) are visible (Figure 6d and Supplementary Figure 2c). Unlike the Claus and Corndog CFP, which form a three-finger claw in the homotrimer formation, the Mysterious CFP trimerizes into a stalk. In this stalk, the N-terminal α-helix forms a trimeric coiled-coil structure followed by four short strands (residues 33–35, 38–42, 49–52, and 57–59), which also trimerize into a compact, β-sheet-rich core. The N-terminal gp30 stalk is inserted into and seals the central cavity of the BHP trimer (Supplementary Figure 5c and 12). The interaction between the BHP and CFP is stabilized by a network of electrostatic, hydrogen-bonding, and hydrophobic contacts that collectively lock the CFP into a conserved surface groove on the BHP (Supplementary Figure 13 and Supplementary Table 5). The CFP engages the BHP groove primarily through its helical segment, which is deeply embedded within a complementary pocket formed by residues from domains I and II of the BHP. Multiple salt bridges anchor the interface, including interactions between CFP R8 and D22 with E316, K172 and D168 of the BHP. Additionally, the CFP R31 hydrogen bonds with BHP T161, providing strong electrostatic stabilization. These polar interactions are further reinforced by hydrophobic residues from the CFP that pack tightly against a nonpolar patch including V175 of the BHP, contributing to shape complementarity and burial of the interface. Together, these cooperative interactions create a stable and well-defined BHP–CFP interface, positioning the CFP securely within the baseplate assembly (Supplementary Figure 12 and 13 and Supplementary Table 5). It is also worth noting that Mysterious gp31 and gp32, annotated as minor tail proteins, are absent from our cryo-EM structure. Both proteins are predicted to contain polyglycine-rich domains (PGDs), which may mediate interactions with lipid moieties [36]. In Bxb1, PGDs are associated with the CFP [8], but this region is not resolved in our structural studies, likely due to the intrinsic flexibility of the Mysterious CFP (Supplementary Figure 2c).

## Discussion

Recent advances in cryo-EM have enabled the structure determination of entire mycobacteriophages at atomic resolution [7, 8], providing an unprecedented opportunity to investigate structural diversity beyond genomic classification. To date, approximately 15,000 mycobacteriophages have been isolated, and nearly 2,800 have been sequenced (https://phagesdb.org/ as of April 2026). Based on nucleotide sequence similarity and gene content comparisons, mycobacteriophages are classified into 37 clusters and six singletons, the latter representing mycobacteriophages without identifiable close relatives [12–14]. Morphologically, the vast majority of mycobacteriophages are siphophages, exhibiting a characteristic architecture composed of an icosahedral capsid, a connector, a long, flexible tail and a baseplate.

In this study, we demonstrate that baseplates from three distinct mycobacteriophage clusters, Claus (Cluster L2), Corndog (Cluster O) and Mysterious (Cluster B5), adopt distinct inverted crown-, barrel- and cage-like architectures, respectively. Genetic analyses indicate that mycobacteriophage populations form a continuum of genetic diversity with non-uniform representation of different mycobacteriophage types [16, 37, 38]. Thus, although mycobacteriophage genomes are grouped into different clusters, they are not entirely unrelated. Consequently, despite their overall architectural divergence, specific regions of these distinct baseplates are likely evolutionarily related, reflecting a conserved functional design principle that seals the tail tube channel and mediates host interactions [7, 8, 25, 28, 29].

Our comparative structural analyses of these three baseplates reveal that despite displaying considerable sequence divergence, they all share a conserved Baseplate Core Complex assembly composed of tail tube protein (TTP), distal tail protein (DTP), and baseplate hub protein (BHP). These components assemble into a conserved 6:6:3 TTP–DTP–BHP complex that mediates the structural transition from the tail tube into the inverted-cone baseplate with an internal plug that effectively occludes the tail tube channel.

**Figure 7.**
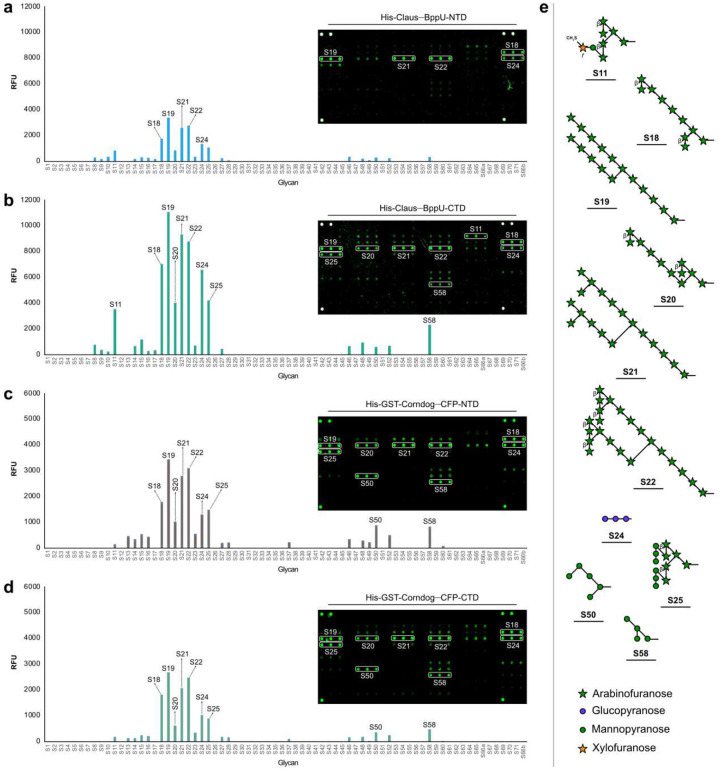
Glycan microarray analysis demonstrates domain-specific glycan binding by Claus BppU and Corndog CFP proteins. Glycan binding specificities of (a) Claus–BppU-NTD, (b) Claus–BppU-CTD, (c) GST-Corndog–CFP-NTD, and (d) GST-Corndog–CFP-CTD were analyzed using a glycan microarray. Bar graphs represent the relative fluorescence intensity (RFU), reflecting the extent of glycan binding across array glycans. Representative fluorescence scans are shown alongside each graph, where bright green spots indicate glycans exhibiting significant binding (e) Schematic structures of representative glycans showing strong binding to BppU and CFP domains. Glycans printed on the array are grouped into major structural classes, including lipoarabinomannan (LAM), capsular α-glucans, phosphatidyl-myo-inositol mannosides (PIMs), phenolic glycolipids (PGLs), and glycopeptidolipids (GPLs), and are labeled according to their array identifiers (S1–S71) [39].

The major structural divergence arises from the BAPs attached to the Baseplate Core Complex. In Claus, a dodecameric BppU ring encircles the DTPs, whereas Corndog attaches six BppU homotrimers to the DTPs. In contrast, Mysterious employs five distinct proteins to form a complex and elaborate assembly around the complex, resulting in its characteristic cage architecture. These BAPs, with various solvent exposed Ig-like domains and carbohydrate binding domains likely serve roles in host cell wall glycan interaction. Supporting this, binding analysis using a mycobacterial cell wall-specific glycan array demonstrates that the Claus BppU and Corndog CFP domains, heterologously expressed in *E. coli*, interact with mycobacterial cell wall glycans (Figure 7) [39]. Similar investigations with Mysterious BAPs were not possible due to our inability to express the proteins. This is consistent with previous observations that the BAPs of mycobacteriophage Douge and Bxb1 interact with host cell wall glycans [7, 8]. Furthermore, our previous cryo-electron tomography studies revealed that baseplates directly contact the mycobacterial cell wall during infection, suggesting that the structural diversity introduced by BAPs is closely linked to host-interaction adaptation [7, 8].

**Figure 8.**
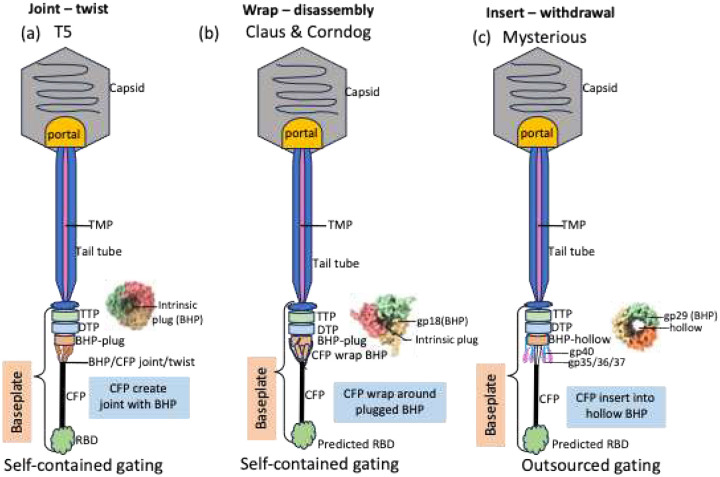
Comparative models of BHP/CFP-mediated gating in T5, Corndog/Claus and Mysterious. Schematic comparison of BHP unlocking mechanism. (a) T5: The BHP lumen is sealed by an internal α-helical plug. CFP binding at the BHP β-sheet domain and receptor engagement drive lateral CFP shift, pulling the internal plug downward to open the lumen. (b) Claus/Corndog: The BHP contains an internal plug reinforced by CFP wrapping around the BHP surface. Channel opening requires sequential release of CFP followed by plug retraction. (c) Mysterious: The BHP lacks an intrinsic plug, and the channel is sealed by structurally similar plugs from CFP. Upon receptor engagement, the CFP plug rotates within the cavity, resulting in its withdrawal and the formation of a DNA-conductive channel. Receptor recognition and conformational changes in gp29/gp30, supported by surrounding accessory proteins gp35/gp36/gp37 (pink blob) and gp40 (cyan line), are proposed to reposition or withdraw the CFP, thereby initiating genome ejection. These models illustrate three distinct gatekeeping strategies: (i) joint/twist BHP unlock (T5), (ii) wrap/disassemble BHP unlock (Claus/Corndog), and (iii) insert/withdrawal BHP unlock (Mysterious), that converge on the common function of controlling DNA ejection. Abbreviation: tail tube protein (TTP), distal tail protein (DTP), baseplate hub protein (BHP), tape measure protein (TMP), central fiber protein (CFP) and receptor binding domain (RBD).

Beyond revealing new aspects of baseplate architectural diversity, this study also uncovers different modes of CFP organization that may be associated with distinct baseplate opening mechanisms for viral genome ejection upon host interactions. Both differ from that used by the well-studied *E. coli* phage T5 [25]. Claus and Corndog employ CFPs that are anchored externally to the BHPs through wrapping interactions, while the enclosed BHPs occlude the tail tube channel through which the DNA would pass upon infection. On the other hand, the Mysterious CFPs achieve the same purpose by inserting into the BHP lumen, suggesting an exogenous plug-like gating mechanism (Figure 8 and Supplementary Figure 4 and 5). Despite differences in whether the plug is endogenous or exogenous, Claus, Corndog, and Mysterious share a conserved three-helix bundle as the BHP plug, indicating that this helical plug may play a fundamental and indispensable role in mycobacteriophage baseplate assembly or host infection (Supplementary Figure 4).

In the *E. coli* phage T5, the trimeric BHPs also harbor an intrinsic α-helical plug that occludes the lumen, functionally similar to the endogenous plug observed in Claus and Corndog (as well as Bxb1 and Douge), [7, 8, 25]. However, in T5, an extended β-sheet domain of the BHP beneath the plug is mechanically coupled to the CFP through a joint-like interface (Figure 8a), that is missing in the mycobacteriophages studied to date. Upon receptor binding, the T5 CFP does not detach from the hub; instead, it undergoes a coordinated sideways displacement together with the BHP extended β-sheet domain. This movement draws the intrinsic α-helical plug downward, resulting in a transition from the enclosed hub into an open conduit while maintaining CFP–BHP contact, thereby enabling phage genome ejection [25]. A similar mechanism has also been reported in bacteriophage λ [28], suggesting conservation of baseplate opening and infection triggering among phages infecting Gram-negative bacteria [22].

Our previous cryo-ET work revealed that mycobacteriophages Bxb1 (Cluster A2) and Douge (Cluster L4), employ a baseplate opening mechanism in which the CFP is fully detached from the BHP during genome ejection [7, 8], which is distinct from those proposed for T5 and λ [25, 28]. In Bxb1, Douge, Claus and Corndog, the CFP does not form a mechanical joint with the BHP. Instead, it forms a sheath around the BHP external surface, potentially acting as a circumferential brace. In the closed state, this sheath may stabilize the enclosed BHPs and indirectly keep the endogenous BHP plug in position (Figure 8b and Supplementary Figure 4 and 5). Receptor binding may impose shear and peeling forces on the CFP sheath, leading to its release. Loss of this bracing may initiate the BHP plug to hinge or retract, thereby opening the BHP lumen. Thus, baseplate opening mechanisms in Bxb1, Douge, Claus and Corndog likely follow a two-step process: initial release of the CFP sheath, followed by conformational changes of the newly revealed BHPs. On the other hand, we propose that Mysterious exhibits a fundamentally different mechanism (Figure 8c and Supplementary Figures 4 and 5). In Mysterious, the BHP lumen is hollow in the closed state and lacks an endogenous plug. Instead, closure is achieved by CFP insertion into the BHP cavity, resulting in CFP functioning as an exogenous plug (Supplementary Figure 13). Receptor binding likely causes the CFPs to rotate within the cavity. This motion may radially de-stabilize BHP–CFP interactions, leading to CFP withdrawal from the cavity and opening the BHP for the genome ejection. Opening of the channel in Mysterious would therefore be a single cooperative step in which the CFP acts as the gate; removing it leads to the BHP opening. Overall, we propose that, to date, two mechanistic variants of CFP-detached BHP opening can be defined: a wrap–disassemble mechanism and an insert–withdrawal mechanism (Figure 8b and 8c), both of which differ from the CFP-attached joint–twist BHP opening observed in phage T5 and λ (Figure 8a).

Overall, this work presents mycobacteriophage baseplate structures from three distinct clusters and identified a structurally and functionally conserved Baseplate Core Complex that is decorated with diversified BAPs that likely contribute to host interactions. Furthermore, we propose two new and distinct baseplate opening mechanisms based on different modes of CFP attachment. Additionally, our atomic-resolution structures provide a structural blueprint for site-directed mutagenesis in these mycobacteriophages, enabling functional validation and mechanistic dissection of CFP detachment-associated baseplate opening. Although additional clusters may exhibit alternative structural variants, this study establishes a foundational framework for understanding mycobacteriophage baseplate assembly and dynamics and informs future efforts in rational mycobacteriophage engineering aimed at expanding host range.

## Materials and Methods

### Phage purification

Mycobacteriophages Corndog was previously isolated and sequenced as reported [13]. Claus and Mysterious were isolated from soil in Taiwan using a previously reported protocol [7, 40]. The phages were purified using the T-streak method, ensuring all subsequent isolates were derived from a single genetic clone. Phage amplification was carried out according to a previously described protocol with a tailored approach to optimize yield [41]. Detailed protocol are described below.

### Host Strain and Growth Media

*Mycobacterium smegmatis* mc2155 was used as the host strain for mycobacteriophage isolation and amplification. For solid culture, the strain was grown on Middlebrook 7H10 agar (HI Media) supplemented with 0.55% (v/v) glycerol, 50 μg/mL carbenicillin (CB; Sigma-Aldrich, USA), and 10 μg/mL cycloheximide (CHX; Acros Organics, USA). For liquid culture, *M. smegmatis* mc2155 was grown in Middlebrook 7H9 broth (BD Difco, USA) containing 40% (v/v) glycerol, 1 mM CaCl_2_ (Acros Organics, USA), 50 μg/mL CB, 10 μg/mL CHX, and 10% (v/v) albumin dextrose complex (ADC). The ADC supplement was composed of 0.53% (w/v) bovine serum albumin (Roche, Switzerland), 0.9% (w/v) NaCl, and 2.1% (w/v) dextrose (Fisher Scientific, USA). Phage isolation and amplification were performed using the double-layer agar method. The bottom agar was prepared using 2.16% (w/v) Middlebrook 7H10 agar supplemented with 0.55% (v/v) glycerol, 50 μg/mL carbenicillin, and 10 μg/mL cycloheximide to support robust mycobacterial growth. The soft top agar overlay was composed of a 1:1 (v/v) mixture of Middlebrook 7H9 broth and MBTA (0.47% (w/v) 7H9 broth with 0.7% (w/v) agar), supplemented with 1 mM CaCl_2_, 50 μg/mL carbenicillin, 10 μg/mL cycloheximide, the *M. smegmatis* host (OD_600_ = 1.5–2.0), and the phage sample. This mixture was poured over the bottom agar layer to allow visualization of phage plaques.

### Phage Isolation

For phage isolation, a moist, warm and aerated soil sample containing decaying organic matter was collected. The isolation procedure was based on the protocol from the Actinobacteriophage database (phagesdb.org), with minor modifications. The soil sample was vigorously mixed with 10 mL of phage buffer (10 mM Tris, 10 mM MgSO_4_, 70 mM NaCl and 1 mM CaCl_2_, pH 7.5). From this mixture, 5 mL of supernatant was collected and filter-sterilized using a 0.22 μm syringe filter. To enhance phage detection sensitivity, the filtered supernatant was combined with 7H9 medium and *M. smegmatis* mc2155 (OD_600_ ≈ 0.6), and the mixture was incubated at 37 °C for 16 h to allow phage amplification. To ensure clonal purity, phages were purified using the T-streak method, ensuring that the isolates originated from a single genetic clone.

### Phage Amplification

Phage amplification was performed following a previously described protocol with minor modifications [41]. A small-scale amplification was first carried out to generate sufficient phage stock for large-scale amplification using the double-layer agar method across 30 plates. After incubation, the plates were resuspended in phage buffer and incubated at room temperature with orbital shaking at 120 rpm for 4 h. To concentrate the phages, the lysate was mixed with 1 M NaCl and 10% (w/v) polyethylene glycol (PEG 8000; Sigma-Aldrich, USA), and the mixture was stirred overnight at 4 °C. Phage particles were then pelleted by centrifugation at 6,400 rpm for 10 min using a HIGH Conic III Fixed Angle Rotor (Thermo Fisher Scientific, USA). The resulting pellets were resuspended in phage buffer (without CaCl_2_) and stirred overnight at 4 °C to ensure complete dissolution. For further concentration and purification, the resuspended phage solution was subjected to cesium chloride (CsCl; Thermo Fisher Scientific, USA) ultracentrifugation using a Type 50.2 Ti Fixed-Angle Rotor (Beckman) at 38,000 rpm for 16 h at 4 °C, yielding high-titer phage lysates. Phage concentration was assessed using spot tests, and the purified high-titer phage stock in CsCl was stored at 4 °C for long-term use.

### Genomic DNA sequencing, assembly, and annotation

Claus and Mysterious genome samples were sequenced using a MiSeq Reagent Kit v2 Micro PE150 (Illumina, USA) in Illumina MiSeq v2 sequencing mode with 300 cycles (pair-end 150 bp) at Academia Sinica High Throughput Sequencing Core of the Biodiversity Research Center. Sequenced raw reads were assembled using GS De Novo Assembler (Newbler) v2.9 (Roche/454 Life Sciences, Branford, CT). Genome annotation was initially performed manually in DNA Master v 5.23.6 (http://cobamide2.bio.pitt.edu) and gene functions were assigned automatically using the BLASTp on DNA Master and subsequently curated manually by using PhagesDB BLASTp,86 HHpred,87 and the PECCAN (http://pecaan.kbrinsgd.org). Transfer ribonucleic acids (tRNAs) were predicted automatically by ARAGORN v1.188 on DNA Master and verified manually by ARAGORN v1.2.38 and tRNAscan-SE v2.0.89. Complete phage genomes were deposited and updated on both PhagesDB (https://phagesdb.org/phages/Corndog/, https://phagesdb.org/phages/Claus/, https://phagesdb.org/phages/Mysterious/) and Phamerator [42].

### Preparation of samples for cryo-EM

A total of 200 μL of CsCl-purified phage stock was dialyzed against phage buffer for a minimum of 2 h at 4 °C prior to grid preparation. For vitrification, 10 μL of the purified phage sample was applied onto glow-discharged Quantifoil holey copper grids (Quantifoil GmbH, Germany) coated with a thin (~2 nm) carbon film. The grids were placed carbon-side down on parafilm in a petri dish and incubated at 4 °C for 15 minutes after being flipped 180°. Subsequently, 4 μL of additional phage sample was applied to the copper side of the grid using a Thermo Scientific Vitrobot Mark IV under controlled conditions (22 °C, 100% humidity). After a 10 sec incubation, excess liquid was blotted away, and the grids were rapidly vitrified by plunging into liquid ethane cooled by liquid nitrogen.

### Cryo-EM imaging and data collection

Cryo-EM grids were screened using a 300 KV Titan Krios microscope at the Academia Sinica Cryo-EM Facility (ASCEM) in Taipei, Taiwan. The microscope is equipped with a K3 summit direct electron detector and a BioQuantum energy filter (Gatan), and operated in super-resolution mode. Automated data acquisition was performed using EPU 2.7.0 following initial grid screening. The energy filter slit width was set to 15eV. Movies were recorded at a nominal magnification of 81,000x, corresponding to a pixel size of 0,5305 Å. A defocus range of −1.0 to −2.2 μm was used and each movie consisted of 50 frames with a total dose of ~50 electrons/ Å^2^.

### Cryo-EM data processing

During cryo-EM data acquisition, all high-resolution movies were motion corrected in parallel using the MotionCorr2 application [43]. As part of this step, a method called two-fold binning was applied. In simple terms, this means combining every block of the four small pixels into one slightly larger pixel, which helps reduce noise and makes the images easier to process. This resulted in a final image resolution where each pixel represented 1.061Å. The image sharpness and contrast were then estimated using CTFFIND4 [44]. Further data processing and analysis of the phage baseplate components were performed by cryoSPARC v4.1 [45].

### Composite cryo-EM maps

Composite maps were generated from the focus-refined maps of the baseplates using UCSF Chimera [46]. To create a unified map, each focus-refined density map was first aligned to its corresponding consensus map. The aligned maps were then combined using Chimera’s “vop max” command, which merges multiple volumes by taking the maximum density value at each voxel position. This approach preserves the strongest signal from overlapping regions and ensures the integrity of high-resolution features from each individual map. Map sharpening and resolution estimation were performed in cryoSPARC v4.1 and the overall resolution was assessed using the Fourier Shell Correlation (FSC) 0.143 criterion. The final 3D density maps were visualized using ChimeraX [47].

### Refinement and model building

The model-building process for the mycobacteriophage Claus, Corndog and Mysterious baseplate structure began with the prediction of individual protein subunit models using AlphaFold2 [48]. These predicted structures were then docked into their corresponding cryo-EM density maps using ChimeraX. In cases where automated docking did not yield optimal fits, manual model building and adjustments were carried out using COOT [49]. The AlphaFold2 predictions for the Mysterious BHP and DTP were ambiguous and could not be unambiguously fit into the cryo-EM density. To overcome this limitation, we employed ModelAngelo for automated model building directly into the experimental map, which enabled reliable tracing of the BHP polypeptide from residues 1–332. The remaining C-terminal ~30 residues, which were not resolved by automated building, were subsequently modeled manually in COOT based on local density features [50]. The aligned and manually adjusted maps were subjected to real-space refinement using PHENIX [51]. All the Figures in the manuscript are drawn by ChimeraX. Comprehensive statistical data, including cryo-EM reconstruction parameters, focus-refined maps, PDB and EMDB IDs and model validation metrics are summarized in Supplementary Table 1 and 6.

### Cryo-EM data collection and image processing for Claus baseplate complex

A total of 15,258 movie stacks of the Claus baseplate complex were collected and processed by motion correction in MotionCor2 and CTF estimation using CTFFIND4, yielding 15,258 micrographs. Particles were automatically picked with Topaz, extracted (box size: 512 → 128), and subjected to 2D classification, resulting in 185,308 particles. Re-extraction with a 512-pixel box size produced 66,544 particles, which underwent ab-initio reconstruction and heterogeneous refinement in C3 symmetry, separating into two classes containing 28.3% and 71.7% (47,725) of the particles, respectively. The larger class was refined non-uniformly with either C6 or C3 symmetry depending on the targeted masked region: Mask1 (top region, C6, 2.61 Å; local C1 refinement to 2.99 Å), Mask2 (middle region, C3, 2.71 Å; local C1 refinement to 2.63 Å), and Mask3 (bottom region, C3, 2.71 Å; local C1 refinement to 2.59 Å), with the resulting local maps combined for the final reconstruction. All refinements were performed in cryoSPARC, and final resolutions were estimated using the gold-standard Fourier shell correlation (FSC) criterion at the 0.143 threshold (Supplementary Figure 14).

### Cryo-EM data processing and reconstruction of Corndog baseplate complex

A total of 6,674 movie stacks were collected for Corndog baseplate imaging. Beam-induced motion was corrected using MotionCor2, and contrast transfer function (CTF) parameters were estimated with CTFFIND4, resulting in 6,674 high-quality micrographs. Particle picking was performed using Topaz, followed by extraction with a 512-pixel box size and Fourier down sampling to 128 pixels, yielding 108,598 particles. Subsequent 2D classification removed poorly aligned particles, retaining 23,182 particles that were re-extracted at the original 512-pixel size. *Ab initio* reconstruction was performed with C3 symmetry, and heterogeneous refinement under C3 symmetry separated two major classes containing 13.1% and 86.9% of particles, respectively. The dominant class underwent non-uniform refinement under both C6 and C3 symmetry, yielding maps at 2.66 Å and 2.74 Å resolution, respectively. To resolve asymmetric features, symmetry expansion was applied separately for the top region (C6 symmetry) and bottom region (C3 symmetry), followed by C1 local refinement with independently generated masks. This yielded final resolutions of 2.70 Å for the top region and 2.66 Å for the bottom region. The locally refined maps were combined to produce the final composite reconstruction of the Claus RBP (Supplementary Figure 15).

### Cryo-EM data processing and reconstruction of Mysterious baseplate complex

A total of 18,782 movie stacks of the Mysterious baseplate complex were collected and processed using MotionCor2 for motion correction and CTFFIND4 for CTF estimation, yielding 18,782 micrographs. Particles were automatically picked with Topaz, extracted (box size: 512 → 128), and subjected to 2D classification, resulting in 433,140 particles. Re-extraction with a 512-pixel box size produced 29,369 particles, which underwent ab-initio reconstruction and heterogeneous refinement in C3 symmetry, separating into four classes containing 51.9%, 20.4%, 12.3%, and 15.4% of the particles, respectively. The largest class (51.9%) underwent heterogeneous refinement to yield a dominant subset (98.7%) processed with non-uniform refinement in C6 symmetry using Mask1 (top region; 2.98 Å), followed by symmetry expansion (C6) and local C1 refinement to 3.21 Å. A small subset (1.3%) from the same branch was refined using a subtraction mask (3.16 Å), followed by particle subtraction, non-uniform refinement, and final reconstruction at 3.39 Å. In parallel, the 15.4% particle class underwent homogeneous reconstruction (14,824 particles), which was refined non-uniformly (C3) into two masked regions: Mask2 (middle region; 3.13 Å, symmetry expansion in C3, local C1 refinement to 3.08 Å) and Mask3 (bottom region; 3.13 Å, symmetry expansion in C3, local C1 refinement to 3.16 Å) shown in Supplementary Figure 16.

### Cloning, expression and protein purification

Claus BppU (gp26) domain 1 residues 1–100 and domain 2 residues118–313 cloned into pET15 His vector. The central fiber protein domains of Corndog, domain 1 (residues 157–323) and domain 2 (residues 440–626), were cloned into the pET15 His-GST expression vector. All constructs were transformed into *E. coli* BL21 (DE3) cells. Cultures were grown at 37 °C in LB medium supplemented with 100 mg l^−^^1^ ampicillin and 50 mg l^−^^1^ kanamycin to an OD600 of 1.2–1.6. Protein expression was induced with 0.5 mM IPTG, followed by overnight incubation at 16 °C. Cells were harvested by centrifugation at 4,000 rpm for 10 min and resuspended in lysis buffer (100 mM phosphate buffer, pH 8.0, 500 mM NaCl, 10 mM β-mercaptoethanol, 10% glycerol, and 25 mM imidazole). The suspension was lysed by sonication and clarified by centrifugation at 18,000 rpm for 20 min at 4 °C. The supernatant was applied to a nickel-affinity column pre-equilibrated with wash buffer (50 mM Tris, pH 8.0, 500 mM NaCl). Bound proteins were washed with buffer containing increasing concentrations of imidazole and eluted with 100–500 mM imidazole. Eluted fractions were further purified by size-exclusion chromatography in buffer containing 20 mM HEPES (pH 7.1) and 150 mM NaCl. Purified proteins were concentrated to ~10 mg ml^−1^ and stored at −80 °C until further use.

### Glycan microarray screening, scanning and data analysis

Glycan microarray assays and analysis were performed as described previously [7, 8]. Briefly, slides were pre-hydrated, blocked with TMST–BSA, and incubated with protein samples (100 μg ml^−^^1^) for 2 h at room temperature. After washing, bound proteins were detected using anti-His primary and Cy3-conjugated secondary antibodies. Slides were washed, dried by centrifugation, and scanned for fluorescence. Microarrays were scanned at 10 μm resolution using a GenePix 4000B scanner (Molecular Devices) with Cy3 detection at 532 nm. Spot intensities were quantified using GenePix Pro 7 software, and relative fluorescence units were obtained by subtracting local background. Mean values were calculated from triplicates using Microsoft Excel.

## Supplementary Material

Supplementary Files

This is a list of supplementary files associated with this preprint. Click to download.

- TableS1S2S6EMDB04212026.docx
- TableS4Corndog0302.docx
- TableS3claus0302.docx
- TableS5mysterious0421.docx
- Figuressupplement04292026.pdf

## Figures and Tables

**Table 1.** Structural organization and comparison of baseplate proteins among phages Claus, Corndog, and Mysterious.

Protein	Claus			Corndog			Mysterious		
	Gene product	oligomer	Resolved	Gene product	oligomer	Resolved	Gene product	oligomer	Resolved
TPP	gp13	6x	1–220 (299)	gp49	6x	1–271 (271)	gp20	6x	13–278 (278)
DTP	gp17	6x	1–303 (303)	gp58	6x	13–583 (583)	gp28	6x	4–477 (477)
BHP	gp18	3x	1–575 (577)	gp59	3x	1–578 (578)	gp29	3x	1–362 (362)
BppU	gp26	6–2x	1–313 (313)	gp60	6–3x	1–282 (282)	–	–	–
CFP	gp20	3x	1–70 (598)	gp61	3x	6–93 (836)	gp30	3x	1–63 (753)
TMP	gp16	–	None (1700)	gp57	–	None (1461)	gp27	–	None (1997)
Inner tail fiber	–	–	–	–	–	–	gp35 gp36 gp37	6–3x	1–209 1–203 1–206
Outer tail fiber							gp40	6–3x	1–136 (136)
Inner tail fiber	–	–	–	–	–	–	gp41	6x	1–378 (378)

The table summarizes the identified baseplate components for the purified bacteriophages Claus, Corndog, and Mysterious, including the corresponding gene products, oligomeric states, and regions resolved in the cryo-EM structures. Baseplate proteins are grouped according to functional modules: tail tube protein (TTP), distal tail protein (DTP), baseplate hub protein (BHP), upper baseplate protein (BppU), central fiber protein (CFP), tape measure protein (TMP), and tail fiber proteins. For each phage, the gene product (gp) designation is listed along with the experimentally determined oligomeric symmetry (e.g., 3×, 6×, 6–2×, or 6–3× copies per complex). The resolved column indicates the amino acid residues modeled in the final structural reconstruction, with total protein length shown in parentheses. A dash (–) indicates absence of an identifiable homolog or unresolved component in the reconstruction, whereas “none” indicates that structure is not resolved, likely due to flexibility or disorder.

## Abbreviations

TTP: tail tube protein
DTP: distal tail protein
BHP: baseplate hub protein
BAP: baseplate accessory protein
BppU: upper baseplate protein
CFP: central fiber protein
